# Supplementary material for: Why are so many pregnancies still unintended in Ghana? A closer look at factors influencing reproductive autonomy
Source: PLOS Glob Public Health. 2026 Jan 22;6(1):e0005850. doi: 10.1371/journal.pgph.0005850 (PMC12826472; doi:10.1371/journal.pgph.0005850)
Supplement: Attachment — Submitted filename: Response to Reviewer.docx [file pgph.0005850.s002.docx]

**RE; Why are so many pregnancies still unintended in Ghana? a closer look at factors influencing reproductive autonomy**

**Point-to-point response to reviewer’s comments**

**Reviewers' comments:**

This is a well-written paper that uses a nationally representative dataset to examine unintended pregnancy among sexually active women in Ghana. The topic is important and the statistical methods are sound. However, the novelty claim is not fully convincing because many of the identified predictors are consistent with prior research among the general reproductive-age population. The authors could strengthen their manuscript by more clearly articulating how their subgroup focus yields new insights, by comparing effect sizes and patterns with broader-population studies, and by exploring potential interaction effects. Below are section-specific comments.

Response: Thank you very much for taking time off your busy schedule to review our study and for acknowledging its relevance. We really appreciate your time and feedback.

Introduction

The research gap presented in lines 75–81 is weak and does not convincingly justify the novelty of focusing on sexually active women. You can make this section stronger by incorporating responses to the following:

1. Can you cite specific Ghanaian or sub-Saharan African studies that have aggregated sexually active women with the broader reproductive-age group and explain how this might have masked important differences?

Response: Thank you very much for pointing this to us. We agree that these lines could be strengthen further. As such, we have provided studies that both aggregated and disaggregated the prevalence of unintended pregnancy across the broader reproductive-age group and sexually active women respectively. We have also explained the gaps that exist in this aggregation and the relevance of disaggregation in the revised manuscript.

2. Are there studies from other contexts that have shown different determinants for sexually active women compared to the broader group? If yes, why do you expect similar or different patterns in Ghana?

Response: Yes. Prior studies in SSA indicate that predictors of unintended pregnancy differ between sexually active women and the general reproductive-age population. In Ghana, we expected similar determinants but stronger effects among sexually active women, given their direct exposure to pregnancy risk, reduced contraceptive negotiation power, and sociocultural barriers affecting consistent contraceptive use and partner communication.

3. How much might the prevalence of unintended pregnancy be underestimated when all reproductive-age women (including those not sexually active) are analyzed together?

Response: Thank you for bringing this to our attention. We have expanded the introduction section in the revised manuscript to capture these dynamics and explain how including non-sexually active women in previous analyses may have led to underestimation of unintended pregnancy prevalence.

4. What specific new insights do you expect this subgroup analysis to reveal that could not be captured by previous broad-population studies?

Response: We appreciate your comment. The revised manuscript explained that this subgroup analysis is expected to reveal more precise behavioral and contextual predictors of unintended pregnancy that are often masked when non-sexually active women are included in broader analyses.

5. How would identifying determinants in this subgroup change the design or targeting of Ghana’s reproductive health programs?

Response: Thank you for this great question. We have now explained the contribution of this study’s subgroup on Ghana’s Family Planning 2030 initiative which emphasizes equity and precision in targeting subgroups most at risk of poor reproductive outcomes.

6. What specific new insights do you expect this subgroup analysis to reveal that could not be captured by previous broad-population studies?

Response: We appreciate this great thought. Specifically, the study contributes new insights to the Ghana’s Family Planning 2030 initiative that was recently launched in Ghana. We believe that by identifying predictors of unintended pregnancy among this population, program managers can more effectively allocate resources toward interventions that address specific barriers within this subgroup.

7. How would identifying determinants in this subgroup change the design or targeting of Ghana’s reproductive health programs?

Response: Thank you. We have previously addressed this comment above. We believe it has been repeated accidentally.

8. Why is the 2017 GMHS particularly suitable for answering this question now, does it contain variables or a sample structure that earlier datasets lacked?

Response: Thank you very much for bringing this to our attention. The 2017 GMHS is particularly suitable for this study because despite being the most recent and comprehensive national dataset on maternal and reproductive health, it has not previously been used to examine unintended pregnancy among sexually active women in Ghana. Using this dataset therefore provides new analytical value and fills an existing evidence gap in the literature.

9. How exactly are you defining this group in the context of your analysis (e.g., past month, past three months, past year), and why is that definition important for understanding unintended pregnancy risk?

Response: We appreciate your comment. Sexually active women were defined as those who have ever had sexual intercourse, based on the GMHS operational definition. This definition is important because it captures all women biologically exposed to pregnancy risk and ensures adequate sample size for national-level analysis, while maintaining consistency with demographic and reproductive health research standards.

10. How does your focus on sexually active women intersect with other high-risk groups, such as adolescents, unmarried women, or rural populations, and why not focus on those instead?

Response: Thank you for this insightful comment. We sincerely appreciate it. While adolescents, unmarried women, and rural populations are indeed recognized as high-risk groups, many of them are already represented within the subgroup of sexually active women, which forms the main focus of this study. Nonetheless, we acknowledge the value of this suggestion and will consider conducting subgroup-specific analyses in future research to further explore these distinctions.

Methods

1. Please clarify the operational definition of “sexually active women” in the GMHS, including the recall period, and discuss potential implications for the prevalence estimate.

Response: Thank you for bringing this to our attention. We have now included the operational definition of sexually active women in the methods section. We have also discussed the potential implications for the prevalence estimate.

2. The exclusion criteria (missing responses, “don’t know” answers, and those never sexually active) removed a large proportion of the original sample; please assess and discuss the potential for selection bias.

Response: Thank you for your comment. We totally agree with you. In the revised manuscript, we have assessed and discussed the potential for selection bias in the methods.

3. The inclusion of independent variables only if they were significant at p≤0.05 in bivariate analysis may omit important confounders; consider including variables based on theoretical relevance as well.

Response: Thank you very much for this comment. We stated in the Methods section under the ‘Independent variable’ subsection that variable selection was guided not only by statistical significance but also by empirical relevance drawn from existing literature. This ensures that all conceptually important confounders were included in the initial analysis.

4. Confirm that the complex survey design and clustering were fully accounted for in both descriptive and regression analyses.

Response: Thank you very much. We have clarified in the Statistical analysis subsection that the complex survey design, including clustering, stratification, and sampling weights was fully accounted for using the ‘svyset’ command in STATA to ensure nationally representative and unbiased estimates.

5. Please explain the rationale for not including variables such as contraceptive use, marital status type, or partner’s fertility preferences, which are known predictors of unintended pregnancy.

Response: Thank you for this insightful comment. We acknowledge that contraceptive use, marital status type, or partner’s fertility preferences are truly known predictors of unintended pregnancy. However, in this study’s analysis, these variables were initially considered but were excluded from the final model due to multicollinearity. Retaining them would have distorted model stability and inflated standard errors. The remaining variables sufficiently captured the underlying constructs these predictors represent.

Results

1. Many associations (age, education, region, cohabitation) are well-established in the literature. Please highlight any differences in magnitude or direction when compared to findings for the broader reproductive-age group in Ghana or SSA.

Response: Thank you very much. We appreciate this insightful comment. Following the reviewer’s suggestion, we conducted an extensive literature appraisal to compare our findings with previous studies. We observed that most existing research on unintended pregnancy in Ghana and sub-Saharan Africa merged pregnant women and all women of reproductive age into a single analytical population, without providing a clear conceptual distinction between these subgroups. As a result, it is challenging to make direct comparisons in terms of effect magnitude or direction, since the population dynamics and exposure risk differ substantially. Most importantly, we want to avoid misinterpretation of these studies.

2. The high prevalence in the Savannah region (86.3%) is striking; please provide sample size and confidence intervals to assess precision.

Response: Thank you for bringing this to our attention. We have now provided sample size and confidence intervals for the Savannah region to assess precision.

3. Internet use shows an interesting reversal from crude to adjusted associations; please explore possible confounding or suppression effects.

Response: Thank you for your comment. In the unadjusted model, internet users appeared to have higher odds of unintended pregnancy, possibly reflecting their concentration in urban, more educated populations with higher reporting rates. However, after adjusting for sociodemographic factors, internet use showed a protective effect suggesting that access to online reproductive health information may enhance awareness and reduce unintended pregnancy risk. We have elaborated on these possible explanations and supporting evidence from the literature in the discussion section.
