# Supplementary material for: Why are so many pregnancies still unintended in Ghana? A closer look at factors influencing reproductive autonomy
Source: PLOS Glob Public Health. 2026 Jan 22;6(1):e0005850. doi: 10.1371/journal.pgph.0005850 (PMC12826472; doi:10.1371/journal.pgph.0005850)
Supplement: Attachment — Submitted filename: Point-to-point response.docx [file pgph.0005850.s003.docx]

**RE; Why are so many pregnancies still unintended in Ghana? a closer look at factors influencing reproductive autonomy**

**Point-to-point response to reviewer’s comments**

**Reviewers' comments:**

1. Overall Assessment

This manuscript examines the prevalence and determinants of unintended pregnancy among sexually active women in Ghana using the 2017 Ghana Maternal Health Survey. Focusing exclusively on sexually active women is a notable strength and adds nuance to existing literature that often aggregates all women of reproductive age. The topic is timely, relevant, and important for public health, reproductive autonomy, and policy targeting. The manuscript is generally well-structured and addresses a significant gap by focusing on a subgroup at genuine biological risk of pregnancy. The statistical methods are mostly appropriate and well presented. However, to strengthen the scientific rigour and the interpretability of findings, several primary clarification and methodological issues must be addressed.

Response: Thank you very much for taking time off your busy schedule to review our study and for acknowledging its relevance. We really appreciate your valuable time and feedback.

2. Comments

Study Design & Methods

The manuscript requires clarification regarding key methodological choices. First, excluding all “don’t know” responses and missing values resulted in the removal of approximately 16,000 respondents, leaving only 1,453 participants; this substantial reduction raises concerns about potential selection bias and warrants justification for whether these exclusions may have systematically affected prevalence estimates. Second, the outcome definition for unintended pregnancy relies solely on responses from women who have ever been pregnant, yet the denominator appears to include women who may have never been pregnant, which could underestimate the proper proportion of unintended pregnancies; therefore, the authors should clarify whether nulligravid but sexually active women were excluded from the analysis or specify how they were accounted for.

Response: Thank you for this important comment. We acknowledge that excluding “don’t know” responses and missing values resulted in a substantial reduction of the analytic sample. This decision was taken to ensure internal consistency and reliable estimates across all variables included in the multivariable models. In the DHS dataset, a large proportion of the excluded cases had missing or “don’t know” responses specifically for core variables used in our estimation. Including these incomplete cases would have produced different denominators across variables and compromised the comparability of estimates in the regression analyses.

About potential selection bias, we conducted a descriptive assessment of the excluded cases and found that the distribution of key background characteristics did not differ meaningfully from those retained in the analysis. This means that the exclusions were unlikely to systematically distort the prevalence of unintended pregnancy.

Regarding the outcome variable, unintended pregnancy is defined in the DHS as: *“When you became pregnant, did you intend to get pregnant at that time?”* This question applies only to women who have ever been pregnant. Therefore, for methodological accuracy, women who were nulligravid regardless of sexual activity were excluded from the denominator, as unintended pregnancy cannot be meaningfully assessed among them. Our analytic sample therefore consisted of sexually active women who had ever been pregnant, including both currently and previously pregnant women. This approach avoids underestimation of unintended pregnancy that would occur if nulligravid women were retained in the denominator. We agree that restricting the analysis to ever-pregnant, sexually active women reduced the sample size, but it ensured that the denominator appropriately reflected the population for whom unintended pregnancy could be genuinely measured.

Data Analysis & Results

The manuscript would benefit from clearer methodological and presentation improvements. Some subgroups, such as women aged 45–49 or those whose first sexual intercourse occurred at age ≥25, produce vast confidence intervals due to very small sample sizes; the authors should either collapse these categories or acknowledge the resulting instability. The multicollinearity assessment also needs clarification. The reported VIF of 1.44 is unclear; please specify whether this represents the mean VIF. Additionally, the variable on internet use shows inconsistent patterns: higher unintended pregnancy in bivariate analyses but a protective association after adjustment. This discrepancy warrants further interpretation.

Response: Thank you very much for these insightful comments. We appreciate your careful attention to the methodological details. In response, we acknowledge that certain subgroups had small cell counts, resulting in wide confidence intervals and unstable estimates. In the revised manuscript, we have clearly noted this limitation in the strengths and limitations section particularly because of the structure of the DHS coding, we needed to retain these age categories for comparability with previous studies.

We also confirm that the reported VIF of 1.44 represents the mean VIF across all explanatory variables included in the multivariable model. This has now been clarified in the Methods section of the revised manuscript.

Concerning the variable on internet use, we agree that the pattern observed requires further explanation. In the revised discussion, we expand on this by explaining that the crude association likely captures confounding factors that have greater access to the internet but may also have higher baseline risk of unintended pregnancy. After adjusting for these covariates, the protective association likely reflects the independent effect of internet exposure on the outcome.

Interpretation & Discussion

The discussion section requires several refinements to ensure accuracy and depth of context. First, causal language should be avoided given the cross-sectional design; for example, statements implying that “internet use reduces unintended pregnancy” should instead state that internet use is “associated with lower odds.” Second, the manuscript relies heavily on comparisons with Ethiopia, Nigeria, Bangladesh, and Indonesia but does not adequately situate the findings within Ghana’s specific reproductive health context, including its policy environment, cultural norms, and health system structure. Third, the notably significant adjusted effect observed in the Savannah region (aOR ≈ 5.5) warrants a more thorough contextual explanation, potentially relating to factors such as nomadic or hard-to-reach populations and cultural norms. Finally, several claims, particularly those concerning sexual education, the influence of digital platforms like TikTok, Snapchat, and Instagram, and gaps in the health system, require proper citations to support them.

Response: Thank you very much for these important observations. We appreciate your detailed guidance on strengthening the discussion section. We fully agree that causal language is inappropriate given the cross-sectional design. In the revised manuscript, all statements implying causality have been corrected to reflect associations rather than causal effects. We also acknowledge the over-reliance on comparisons with Ethiopia, Nigeria, Bangladesh, and Indonesia. We have now substantially revised the discussion to foreground Ghana’s reproductive health context.

Furthermore, we agree that the strong adjusted effect observed for women in the Savannah Region needs deeper contextual exploration. In the revised manuscript, we discuss plausible contextual explanations such as limited health service availability in hard-to-reach and nomadic communities, higher levels of poverty and illiteracy, patriarchal decision-making norms, and persistent gaps in contraceptive access and counselling in northern Ghana.

We really appreciate the reminder regarding sources. In particular, all claims related to sexual education, the influence of digital platforms have now been supported with appropriate citations from peer-reviewed literature.

Novelty & Contribution

The study’s focus on sexually active women is clearly articulated and adds meaningful value. However, the novelty claim would be more compelling if the authors explicitly compared their findings with studies that analyze the whole reproductive-age population and demonstrated how limiting the sample to sexually active women affect prevalence estimates or effect sizes.

Response: Thank you very much. We really appreciate this insightful comment and totally agree with you. Following your brilliant suggestion, we conducted an extensive literature appraisal to compare our findings with previous studies that analyzed the whole reproductive-age population. We observed that most existing research on the topic merged pregnant women and all women of reproductive age into a single analytical population, without providing a clear conceptual distinction between these subgroups. More specifically, you would find that studies that indicated women of reproductive age as their study population actually interviewed pregnant women. As a result, it is challenging to make direct comparisons particularly in terms of effect magnitude or direction, since the population dynamics and exposure risk differ substantially. Most importantly, we want to avoid misinterpretation of these studies.
