# Supplementary material for: Why are so many pregnancies still unintended in Ghana? A closer look at factors influencing reproductive autonomy
Source: PLOS Glob Public Health. 2026 Jan 22;6(1):e0005850. doi: 10.1371/journal.pgph.0005850 (PMC12826472; doi:10.1371/journal.pgph.0005850)
Supplement: Attachment — Submitted filename: Point-to-point_response_auresp_3.docx [file pgph.0005850.s004.docx]

**RE; Why are so many pregnancies still unintended in Ghana? a closer look at factors influencing reproductive autonomy**

**Point-to-point response to reviewer’s comments**

**Reviewers' comments:**

1. Reviewer #2: SECOND-ROUND REVIEWER REPORT

The manuscript has improved substantially in clarity, contextualization, and justification of the analytical focus on sexually active women. Several of the original concerns have been adequately addressed. The revised manuscript clearly justifies the restriction of the analysis to sexually active women, strengthening its overall contribution, and now transparently reports the scale and reasons for sample exclusions while appropriately acknowledging the potential for selection bias, even though additional supplementary comparisons could further enhance transparency. The authors have improved the interpretation of internet use by addressing discrepancies between crude and adjusted associations and supporting this Discussion with relevant literature. The Discussion is better contextualized within Ghana, incorporating policy, regional, and health system perspectives, particularly for the Savannah region, and the authors have avoided causal language while clearly articulating the study’s novelty relative to prior analyses of the general reproductive-age population.

Recommendation

The manuscript shows strong potential, has improved meaningfully, and can be considered for publication.

Response: We sincerely thank Reviewer #2 for the thoughtful second-round review and the positive assessment of the manuscript’s improvements. We are grateful for the encouraging recommendation and constructive guidance that further strengthened this work. Thank you.

2. Comments

Abstract

Line 30 – Change 'The study concluded' to 'The study concludes'.

Response: Thank you for pointing this out. This change has been affected in the revised manuscript.

Introduction

Line 70 – Revise as 'The extant literature has…'

Response: Thank you for this suggestion. This change has been affected in the revised manuscript.

Line 71 – Move the references (5,11) to the end of the sentence with the other references.

Response: Thank you for your comment. The references have been moved to the end of the sentence as suggested.

Lines 75-90 - These paragraphs provide a good indication of the gap, however, based on your small sample size, readers need to be sure about who exactly was selected for your sample. Is it limited to currently pregnant or ever pregnant women? If so, indicate the variable used to filter other women out. Then let the exact target population reflect in the paragraphs, in that not only does your study limit the sample to sexually active women, it limits it further to those currently/ever pregnant. Ensure that the specific population is discussed and this runs throughout the paper.

Response: Thank you very much for this important comment. In response, we have revised the manuscript to clearly specify the exact analytical population given the reduced sample size. Additional clarification has been provided in the Study population, and Sampling strategy and sample size sections to explain the variables used to arrive at the final sample. We have now explicitly stated that the analysis was restricted to sexually active women who were pregnant at the time of the survey, consistent with the GMHS skip pattern for the unintended pregnancy variable, and that women who reported never having had sex were excluded using the age at first sexual intercourse variable. Based on these revisions, “sexually active women who were pregnant at the time of the survey” was used at the first definition after which “currently pregnant women” was used consistently throughout the manuscript.

Methods

Lines 130-131 - again, provide clarity on which variables you used to exclude women due to their missing and don't know responses. If it is the currently or ever pregnant variable? If so, then indicate this. An unweighted frequency of the age at first sex variable indicates 3,665 had never had sex, 5 refused to answer and over 21,000 had ever had sex. If you are defining those sexually active as those who had sex in the past year or month, and thus filtered many women out, then this needs to be clear.

Response: Thank you for the opportunity to clarify this point. We confirm that the unintended pregnancy question in the 2017 GMHS was administered only to women who were pregnant at the time of the survey, consistent with the GMHS questionnaire skip pattern. As a result, only 1,747 women responded to the unintended pregnancy item. The substantial reduction in the analytical sample therefore reflects the restriction of the outcome variable to currently pregnant women, rather than filtering based on recent sexual activity or ever-pregnancy status. After excluding respondents with missing data on the unintended pregnancy variable and those who reported never having had sex, the final analytical sample comprised 1,453 sexually active women who were currently pregnant and provided complete information on pregnancy intention. We have revised the Methods section to explicitly state this and ensure consistency throughout the manuscript.

Line 152 - tell us which regions were combined in each of the three zones.

Response: Thank you for this comment. We have revised the Methods section to explicitly specify the regions included under each of the three ecological zones.

Results

Check values in Table 1 – used the internet in the last 12 months. The same value has been written twice.

Response: Thank you for pointing out this error. We have reviewed Table 1 and corrected the duplication in the values reported.

Discussion

In the Discussion, the authors can also reflect on how unintended pregnancy is also higher when fertility intention declines among a group - when people want fewer children, they are more likely to declare additional pregnancies as unintended.

Response: Thank you for this insightful suggestion. We agree that unintended pregnancy is influenced not only by contraceptive use and access but also by shifts in fertility intentions. In response, we have expanded the discussion to reflect this.

The authors should check other publications for the in-text citation format which usually includes the author’s last name and the reference – for instance 'Nyarko [24] stated that' or 'Sana [45] found that'.

Response: Thank you for raising this observation. We have revised the manuscript to ensure that all in-text citations are consistently formatted to include the author’s last name followed by the reference number, in line with standard journal practice.
